# Cell competition and cancer from *Drosophila* to mammals

**DOI:** 10.1038/s41389-023-00505-y

**Published:** 2024-01-03

**Authors:** Bojie Cong, Ross L. Cagan

**Affiliations:** School of Cancer Sciences, University of Glasgow, Wolfson Wohl Cancer Research Centre, Garscube Estate, Switchback Road, Bearsden, Glasgow, Scotland G61 1QH UK

**Keywords:** Cell growth, Oncogenes

## Abstract

Throughout an individual’s life, somatic cells acquire cancer-associated mutations. A fraction of these mutations trigger tumour formation, a phenomenon partly driven by the interplay of mutant and wild-type cell clones competing for dominance; conversely, other mutations function against tumour initiation. This mechanism of ‘cell competition’, can shift clone dynamics by evaluating the relative status of clonal populations, promoting ‘winners’ and eliminating ‘losers’. This review examines the role of cell competition in the context of tumorigenesis, tumour progression and therapeutic intervention.

## Introduction

Cancer stands as a primary global contributor to mortality. It arises from aberrant and uncontrolled cellular proliferation, largely triggered by genetic mutations [1, 2]. So far, more than 600 cancer driver genes have been identified; broadly observed drivers include *TP53*, *KRAS*, *Pi3K3CA*, *APC*, *CTNNB1*, *CSMD3*, *FAT1*, *NOTCH1* and *SMAD4* (https://www.intogen.org/search). Recent sequencing efforts have unveiled the widespread presence of cells—often organised as clones—carrying canonical cancer mutations within our tissues such as *TP53*, *FAT1* and *NOTCH1*. Remarkably, many of these clones are functionally and phenotypically normal, with some even undergoing positive selection [3–7].

**Fig. 1:**
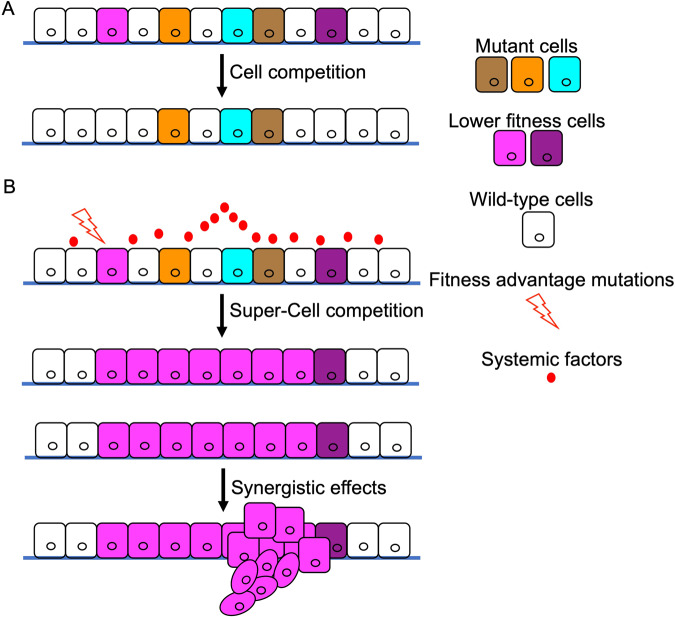
Cell competition and tumour progression. **A** This diagram illustrates the core concept of cell competition within the context of tumour progression. Various clonal variations present in tissues are symbolised by cells of different colours. Specifically, cells with lower fitness levels, denoted by magenta and dark purple cells, are identified as ‘losers.’ These ‘loser’ cells are eliminated from the tissue by the neighbouring high-fitness ‘winner’ cells. **B** Specific genetic alterations or systemic factors can bestow a fitness advantage to cells that initially possess lower fitness levels. This enables them to evade cell competition and undergo uncontrolled proliferation. These benign tumour cells (magenta) have the potential to turn malignant when they encounter mutated cells (dark purple) capable of promoting tumour cell growth or malignancy.

An increasing number of variants are recognised as capable of initiating changes in cellular functions that spark competition between mutant clones and their wild-type neighbours. Clones with diminished fitness, whether the mutants themselves or their wild-type neighbours, are actively eliminated from the tissue by their more ‘fit’ neighbours. This process, termed ‘cell competition’ represents a specialised manifestation of clone dynamics and cell-to-cell interaction crucial in upholding tissue homeostasis through the removal of lower fitness cells (Fig. 1A).

Cell competition was first observed in 1975 within Drosophila wing discs involving heterozygous loss of a ribosomal gene (*Rp*^*+/−*^) [8]. Despite being viable and forming functional organisms, *Rp*^*+/−*^ cells are selectively eliminated when surrounded by wild-type neighbours [8, 9]. Two decades later, Drosophila cells carrying ‘neoplastic tumour-suppressor’ genes such as *scribble* (*scrib*), *discs large* (*dlg*), *lethal giant larvae* (*lgl*), *rab5*, *vps25*, *Tumor susceptibility gene 101* (*TSG101*), and *avalanche*, were identified as “losers” when juxtaposed with wild-type cells; of note, in isolation these cells displayed over-proliferation [10–19]. These studies suggested the interesting possibility that cell competition plays a role in eliminating transformed cells. On the other hand, Drosophila imaginal disc cells that overexpressed the potent oncogene dMyc exhibited a competitive edge over wild-type cells due to their enhanced fitness, fostering tumour initiation [20, 21]. These ‘dMyc high’ cells were christened ‘super-competitors’. These studies underscore how cell competition can function as both a tumour-suppressive and tumour-promoting mechanism.

Over the past decade, insights gleaned from Drosophila studies have been largely validated in mammalian systems. The principles of cell competition and super-competition have been evolutionarily conserved, playing pivotal roles in eliminating lower fitness cells during embryonic development and maintaining homeostasis in mammalian systems [22]. Importantly, numerous instances of super-competition observed in mammalian systems involve signalling pathways often disrupted in cancer, including the N-MYC, TP53, NOTCH, WNT and HIPPO pathways [23–25].

This phenomenon has spurred increasing interest in understanding the role of cell competition in cancer. Can cell competition explain the existence of phenotypically normal cell clones harbouring canonical cancer mutations in our body? Are neighbouring cell fields responsible for maintaining quiescence of these clones and if so, how?

In this perspective, we delve into our evolving understanding of how cell competition mediates tumorigenesis and tumour progression at the level of clone dynamics. We highlight early Drosophila studies that introduced the concept of cell competition and how more recent studies in mammalian models have validated and expanded on these concepts. Further, we discuss the exciting potential for leveraging cell competition mechanisms for cancer therapy.

## Signals that regulate cell competition

### Control of protein synthesis

The concept of cell competition and ‘loser’ cells were initially observed in cells bearing heterozygous mutations for various Drosophila ribosomal proteins (*Rp*^*+/−*^), which resulted in a subtle decrease in protein synthesis [26]. Cells harbouring mutations in *Helicase25E* (*Hel25E*), also linked to compromised protein synthesis, were similarly eliminated as ‘losers’ [27]. Conversely, even subtle overexpression of dMyc, a factor that promotes protein synthesis in both mammalian cells [28] and Drosophila [27], led to expansion of clones as ‘super-competitors’; this advantage was reversed if ribosomal function was also (subtly) compromised, again emphasising the importance of relative protein synthesis as a local measuring stick [20].

In Drosophila cell competition, regulation of protein synthesis is closely linked with other cellular properties including endoplasmic reticulum (ER) stress. For instance, the transcription factor Xrp1, which contains a basic leucine zipper (bZIP) domain, plays a pivotal role in driving cell competition in *Rp*^*+/−*^ cells [29]. When confronted with wild-type cells, ER stress triggers the upregulation of Xrp1 expression and Perk-mediated phosphorylation of the eukaryotic translation initiation factor 2 (eIF2α). The result is reduced protein synthesis, inhibition of cell proliferation, and induction of apoptosis in *Rp*^*+/−*^ and *Hel25E*^*−/−*^ cells [26, 30–32]. Similarly, mutations in other ER stress-related genes such as *wollknaeuel* (*wol*), *Elongator complex protein 3* (*Elp3*) and *calreticulin* also led to the elimination of cells when confronted with wild-type cells, classic cell competition. Together, these studies suggest that protein synthesis can serve as both a mediator and biomarker of cell competition. This competition may occur at several stages of development: for example, mutations in the ribosomal protein RPL24 trigger competitive interactions among cells in the mouse blastocyst [33].

### Signalling pathways- Hippo

In Drosophila, mutations in Hippo pathway components such as *fat*, *hippo*, *expanded*, *salvador*, *warts* and *mats* trigger a super-competitor state in cells, resulting in the elimination of their wild-type neighbours [34]. These super-competitors achieve this by upregulating expression of dMyc, a process mediated by the transcriptional co-activator Yorkie (Yki), a downstream effector of the Hippo pathway [35]. Similar super-competition driven by HIPPO pathway mutations has been documented in cultured mammalian fibroblasts [36]. These studies suggest that Hippo-mediated super-competition is regulated at least in part by control of protein synthesis in both flies and mammals.

### Signalling pathways- Flower

A key pathway that mediates cell competition through local cell-cell communication in *Rp*^*+/−*^ cells, dMyc cells [37, 38] and *Hel25E*^*−/−*^ cells [27] is the Flower-Azot axis. Flower is a transmembrane protein that is highly conserved among metazoans including humans. The fly genome encodes four isoforms: the two Flower-Ubi are associated with winner status and Flower-Lose-A and Lose-B are linked to loser status. During *Rp*^*+/−*^ or dMyc-induced cell competition, Flower-Lose isoforms are increased specifically in loser cells. This upregulation of Flower-Lose results in increased expression of *ahuizotl* (*azto*), thereby inducing expression of the pro-apoptotic gene *head involution defective* (*hid*) [38]. When Flower-Ubi-expressing cells are co-cultured in direct contact with Flower-Lose-expressing cells, Flower-Lose cells are lost by apoptosis as Flower-Ubi cells expand through compensatory proliferation.

Notably, a subset of human cancers exhibits elevated levels of Flower-Ubi orthologs (hFWE-Win), while adjacent stromal tissues often display substantial upregulation of FLOWER-Lose (hFWE-Lose) isoforms. This phenomenon is more prevalent in malignant tumours than benign ones [39]. Overexpression of hFWE-Win in tumour cells is sufficient to non-autonomously upregulate expression of hFWE-Lose in neighbouring cells; conversely, knockdown of hFWE-Win in tumour cells inhibited tumour overgrowth and metastasis [39]. Together, these findings are consistent with the view that cell competition plays a role in driving progression of at least some human tumours.

### Signalling pathways- NFκB

*Rp*^*+/−*^ cells, dMyc overexpressing cells, and *Hel25E*^*−/−*^ cells all regulate cell competition by modulating activity of the Nuclear Factor-kappa B (NF-κB) signalling pathway. With regards to Drosophila cell competition, the primary upstream regulator of NF-κB signalling is the Toll pathway. Activation of Toll directs formation of a heterodimer consisting of Relish plus Dif or Dorsal, which in turn directs expression of a large panel of target genes [40]. Drosophila has nine Toll-related receptors, Toll-1 to Toll-9. In *Rp*^*+/−*^-mediated cell competition, losers were eliminated through the Toll-3,9-Dif/Dorsal pathway, which triggered expression of the proapoptotic gene *reaper* (*rpr*). In dMyc-driven cell competition, the Toll-2,3,8,9-Relish pathway removed losers (wild-type cells) by inducing the pro-apoptotic gene *hid* [41]; *Hel25E*^*−/−*^-associated cell competition similarly used Dif/Dorsal to activate Hid-mediated apoptosis [27]. Similarly in murine fibroblasts, elevated NF-κB promoted apoptosis by activating TP53 [42].

### Signalling pathways- TGF-ß

In flies, protein synthesis-associated cell competition has also been connected to the competitive ‘capture’ of factors such as the TGF-β orthologue Decapentaplegic (Dpp) [9], as well as cell engulfment through a Draper-Wasp-Phosphatidylserine receptor-Mbc/Dock180-Rac1-mediated network [43]. When Dpp/TGF-β binds its receptor, subsequent phosphorylation of the downstream effector Smad creates a signalling complex that enters the nucleus to activate a panel of target genes [44]. When *Rp*^*+/−*^ cells were surrounded by wild-type cells, this Dpp response was diminished due to upregulation of the downstream transcriptional repressor *brinker* (*brk*); instead, *Rp*^*+/−*^ cells died by JNK-dependent apoptosis. This suggests a model in which neighbouring cells compete for limited survival factors such as Dpp, resulting in the removal of lower-fitness cells.

Taken together, these studies illustrate that cell competition, prompted by variations in protein synthesis among neighbouring clones, is an evolutionarily conserved mechanism likely involved in selecting unfit cells during development. Tumour cells appear to exploit this process, expanding at the expense of neighbouring cells through mechanisms of cell competition. This process is regulated by different factors in different situations, including Flower, NF-κB signalling, and the capture of secreted growth factors such as TGF-β. Many of these factors are closely tied to cancer. For example, TGF-β plays a central role in maintaining cancer stem cells (CSCs), inducing expression of the CSC marker CD133 in liver cancer cell lines to enhance tumorigenesis in mice [45]. These findings have given rise to the idea that tumour cells act as ‘super-competitors’ that can disrupt tissue homeostasis, a concept we explore in the next section.

## Cancer-associated mutations promote tumorigenesis by altering cell competition

The outcome of cellular competition hinges on dynamic processes that continually interact at clone boundaries. A ‘loser’ cell can transition into a ‘winner’ when it acquires oncogenic alterations granting a selective fitness advantage or when neighbouring wild-type cells accumulate genetic variations conferring a selective fitness disadvantage. For example, in Drosophila, ‘loser’ cells experiencing functional loss of genes that regulate apico-basal cell polarity—such as *scribble* (*scrib*) or *discs large* (*dlg*)—can cooperate with oncogenic Ras (Ras^G12V^) or effectors of the Hippo pathway such as Yki. The result is clonal expansion, tumour overgrowth, and metastatic invasion [46]. Alternatively, pairing defective protein trafficking (*e.g*., *rab5*^*−/−*^) with overexpression of the microRNA *bantam* is adequate to drive cells towards tumorous overgrowth and malignancy [47]. Overexpression of dMyc, Notch, or Jak/Stat signalling also can reverse the elimination of *scrib*^*−/−*^ clones [12, 48, 49]. As mentioned above, pathways such as MYC, NOTCH, and HIPPO are frequently elevated in cancer. Perhaps the role of some oncogenes is to rescue cancer cells from loser status in cell competition within a cancerization zone.

### Role for wild-type cells

With respect to cancer, the role of neighbouring wild-type cells can also strongly influence cell competition dynamics and tumour stability. Functional loss of the ligand Sas or Serpin5 (Spn5) in adjacent wild-type cells has the capacity to transform ‘loser’ cells into ‘winners,’ as observed in *scrib*^*−/−*^ cells. Sas is a ligand that binds the receptor tyrosine phosphatase Ptp10D. Typically, Sas/Ptp10D interact at the apical surface of epithelial cells. However, at the interface between *scrib*^*−/−*^ cells and neighbouring wild-type cells, they relocate to the lateral membrane where they engage in trans-interactions. This interaction triggers Ptp10D signalling in *scrib*^*−/−*^ cells, resulting in the suppression of epidermal growth factor receptor (Egfr) signalling and subsequent elimination of *scrib*^*−/−*^ cells. In the absence of Sas-Ptp10D signalling, *scrib*^*−/−*^ clones enhance Egfr and Jnk signalling, which cooperatively activates Yki, leading to overgrowth [50].

Spn5 is a secreted serine protease inhibitor that negatively regulates the Toll ligand Spätzle (Spz). Spn5 mutations in otherwise wild-type cell neighbours activate Toll signalling in *scrib*^*−/−*^ cells, triggering Yki activation and subsequent overgrowth of *scrib*^*−/−*^ cells [7]. Regulators of cell competition through Toll/NF-κB are of particular relevance to cancer: elevated Toll/NF-κB signalling has been observed in various tumour types including breast cancer [51], lung cancer [52], leukaemias [53], and lymphomas [54]. In cholangiocarcinoma, NF-κB signalling promotes progression by regulating cell proliferation, invasion and epithelial-mesenchymal transition (EMT) [55, 56]. Inhibiting NF-κB signalling has demonstrated anti-tumour responses [57, 58], and inhibitors have shown promise in clinical studies, most notably for lung cancer patients [59].

Beside the NF-κB signalling pathway, several other signalling pathways have been identified as used by wild-type cells in their role of eliminating tumour cells. For instance, in the pancreas, KRAS^G12D^ mutant cells are recognised by neighbouring wild-type cells by their increased expression of the membrane receptor EPHA2, which leads to the extrusion of mutant cells from the tissue. In contrast, the absence of EPHA2 causes retention of KRAS^G12D^ clones and promotes the development of pancreatic intraepithelial neoplastic lesions [60, 61]. In the local environment of liver tumours, wild-type hepatocytes exhibit activation of Hippo pathway effectors YAP and TAZ: deletion of YAP and TAZ in these hepatocytes accelerates tumour growth [62]. Intestinal tumours transformed with mutations in *APC*, *KRAS*, and *TP53* have been shown to enhance their growth when interacting with wild-type small intestine cells, providing direct evidence that cellular competition promotes tumour growth [63].

Besides oncogenic mutations directly converting ‘losers’ to ‘winners’, systemic factors also play a significant role in regulating clone dynamics and cell competition in Drosophila. For instance, heterozygosity of the Insulin Pathway effector *chico* in insulin-producing cells (IPCs) results in hyperinsulinemia by upregulating Drosophila insulin Dilp2. This induction leads to insulin/mTOR activation in *scrib*^*−/−*^ cells, enhancing protein synthesis and causing overgrowth [64]. Importantly, high-caloric diets such as those rich in dietary sugar or fat can disrupt the fitness balance between oncogenic cells and neighbouring wild-type cells, ultimately leading to tumorigenesis in both Drosophila and mice [65, 66]. Interestingly, dietary nutrients have been found not only to reverse the ‘loser’ state but also to enhance the aggressiveness of these previously disadvantaged cells. In oral squamous cell carcinomas, dietary palmitic acid, which is a saturated long-chain fatty acid, heightens the metastatic potential of tumour cells by upregulating expression of the fatty acid receptor CD36 [67]. These findings align with clinical observations where obese patients exhibit a higher risk for several cancer types including colorectal, uterine, and postmenopausal breast cancer [68].

In summary, the transition from ‘loser’ to ‘winner’ status can be driven by genetic variants that act autonomously or by modifying the tumour’s environment, thereby promoting tumour progression (Fig. 1B). This accumulation of oncogenic alterations, a hallmark of cancer cells, may in part reflect the interplay between cell competition and the selection for ‘winners’ as a tumour advances.

## Cell competition can drive cancer evolution

The process of cell competition likely plays a significant role in driving the evolution of cancer, alongside its contribution to enhancing tumour progression. Cancer progression, on one level, represents a clonal evolutionary process fuelled by cellular heterogeneity [69]. This phenomenon holds important implications for cancer initiation, advancement, and therapeutic strategies.

Intertumoral cell competition might initially seem counterproductive for tumour progression, as a considerable number of cancer cells are self-eliminated. This occurs because tumour cells frequently display ‘Gompertzian’ growth characteristics in which the doubling times of tumour cells (typically 1-2 days) are significantly faster than the doubling times of tumours themselves (approximately 60-200 days). This reflects the substantial proportion of tumour cells that undergo cell death before they have the opportunity to divide [70]. This in turn leads to competition between transformed cells within the tumour and, in turn, tumour evolution. For example, when human breast Loxl3 subclones are surrounded by parental tumour cells (at a ratio of 1:18), the number of these subclones increases approximately tenfold, albeit failing to promote an overall increase in tumour size [71]. This phenomenon is largely attributed to competition among subclones for limited space and nutrients. Dominant subclones frequently bear selective mutations that result in defects in tumour suppressor genes or an upregulation of oncogenes [72, 73]. In glioblastoma, the heterogeneous expression of the Hippo pathway effector YAP led to cell competition, resulting in elimination of ‘low YAP’ tumour cells by ‘high YAP’ tumour cells; this process facilitated tumour progression [74]. Similarly, agent-based models simulating cell competition within tumours reached the same conclusion [75].

These and similar studies indicate that intertumoral subclones also engage in competition in human cancer, a phenomenon referred to as clonal interference. Of note, however, not all subclones exhibit competitive behaviour; some may have synergistic effects. For instance, in Drosophila, clones of Ras-activated benign tumours underwent transformation into invasive tumours when juxtaposed with clones of *scrib*^*−/−*^ cells through upregulation of Jak-Stat signalling [76]. Similarly, Ras-activated cells juxtaposed with cells with mitochondrial dysfunction [77] or Src-activation [78] also exhibited this transformative synergy. Polyclonal tumours in human cancers are commonly associated with metastasis, whereas monoclonal tumours typically do not exhibit metastatic behaviour [71]. Notably, the evolution of tumour subclones is not monolithic, and parallel evolution is often observed in human patient samples [79–81]. Typically, various subclones of cells display varying degrees of sensitivity to anti-cancer drugs, often leading to resistance to cancer treatments [81, 82].

In summary, tumour cells with high fitness outcompete their lower-fit counterparts, leading to their own expansion by cell competition. This shapes a microenvironment conducive to tumour progression and enhances the chances of highly adaptable tumour cells encountering populations that foster their proliferation or malignancy (Fig. 1B). Disturbing these clonal interactions, such as surgically removing a primary clone, can inadvertently impact interactions within the remaining tumour, with unpredictable results. Thus, comprehending the role of cell competition in cancer evolution is pivotal for devising effective cancer therapies.

## Leveraging cell competition as a therapeutic target in cancer

Tumorigenesis and tumour progression are regulated by both local and systemic factors that impact cell competition. Targeting the underlying mechanisms of cell competition in cancer may offer a promising avenue for novel therapeutic interventions aimed at preventing and suppressing tumours by inhibiting the competitive advantage of oncogenic mutant clones. Indeed, recent work has argued that many tumours contain mutations with a primary role of masking the tumour’s otherwise ‘loser’ nature [83]. In Drosophila, several interventions have been reported. For example, hyperinsulinemia can transform *scrib*^*−/−*^ cells from ‘losers’ into ‘winners’ by upregulating insulin-mTOR signalling, a pathway regulating protein synthesis. However, the antidiabetic drug metformin suppressed tumorigenesis in *scrib*^*−/−*^ cells by downregulating protein synthesis [64]. L-type amino acid transporter 1 (LAT1) inhibitors such as BCH or KYT0353 strongly reduced the dominance of *Ras*^*G12V*^*;scrib*^*−/−*^ tumour cells over wild-type cells by curtailing mTOR signalling [47]. High dietary sugar enhanced the advantage of *Ras*^*G12V*^*;csk*^*−/−*^ cells, leading to their outcompeting wild-type cells by activating Wnt signalling. While individual agents like acarbose (reduces glucose), pyrvinium (Wnt signalling inhibitor) or AD81 (Ras signalling inhibitor) worked poorly, various combinations of these three strongly suppressed high sugar diet-induced tumorigenesis [65].

In mice, there have been notable strategies to disrupt the competitive advantage of mutant clones in different tissue contexts. For instance, in the intestine, enhancing the Wnt pathway in wild-type cells using lithium chloride [84] or blocking the Wnt antagonist Notum [85] effectively nullified the competitive advantage of *APC* mutant clones. In the oesophageal epithelium, exposure to ionising radiation favoured the expansion of pre-cancerous *TP53* mutant clones due to their greater resistance to radiation-induced redox stress compared to their wild-type neighbours. However, antioxidants administered alongside low-dose irradiation improved the fitness of wild-type cells and facilitated elimination of *TP53* mutant clones [86]. As described above, reducing NOTCH1 activity through, *e.g*., a targeted antibody proved protective in mouse oesophageal tumour models. In a mixed-culture model using normal Madin-Darby canine kidney (MDCK) cells, neighbouring wild-type cells increased the expression of *Cyclooxygenase 2* (*COX2*) and pushed oncogenic RAS cells out from the apical surface. Inhibiting COX2 activity in wild-type cells, by *COX2* knockout or the COX inhibitor ibuprofen, significantly boosted the apical extrusion of oncogenic RAS cells. In mouse pancreatic epithelial cells, *COX2* expression was found to be elevated in oncogenic RAS cells; ibuprofen again promoted apical extrusion of oncogenic RAS cells [87]. Additionally, tumour cells with mutations *APC*, *KRAS*, and *TP53* prompted the removal of wild-type small intestine cells by cell competition. Yet, the JNK inhibitor JNK-IN-8 robustly suppressed the elimination of wild-type cells and inhibited tumour growth [63]. Thus, increasing fitness of neighbouring wild-type cells could function as a therapeutic strategy to limit tumour progression.

However, not all emergent mutant clones contribute to tumorigenesis. Jones and colleagues provide an example of how mutant NOTCH1 isoforms are commonly found in normal oesophageal tissue and can act as a preventive factor against cancer [5, 88]. *NOTCH1* was identified from deep sequencing of tissue samples from nine healthy donors to map the clonal structure of the oesophagus. They found that somatic mutations accumulate with age and are caused mainly by intrinsic mutational processes: normal aging human oesophageal epithelium is colonised by clones with biallelic *NOTCH1* mutations that disrupt signalling, affecting up to 80% of cells. These mutations are more frequent than in oesophageal cancers, suggesting they impair carcinogenesis. The authors propose that *NOTCH1* mutations in normal tissue are a consequence of aging and environmental damage, and that they confer a fitness advantage by altering cell fate and differentiation. In the mouse oesophagus, *NOTCH1* wild-type cells are more likely to contribute to tumours than *NOTCH1* mutant cells; *NOTCH1* loss reduced tumour size by slowing cell division and attenuating signalling downstream of mutant ATP2A2. This work highlights the intriguing possibility that some emergent clones are part of a normal defence against tumour progression.

Intertumoral heterogeneity stands out as a significant force behind drug resistance and has the capacity to disrupt clonal evolution, reshaping the fitness environment and guiding the neoplastic cell population along different trajectories. This dynamic shift in local clone dynamics offers an innovative and potentially potent therapeutic approach to mitigate tumour progression, opening new avenues for the prevention and treatment of cancer.

## Conclusions and perspective

Overcoming drug resistance in cancer therapy remains a formidable challenge, despite extensive efforts. Novel approaches are needed and the burgeoning field of cell competition provides us with an especially innovative and promising avenue. This research domain, which originally took root in Drosophila studies, is now at the crossroads of genomics and oncology in human cancer studies, which has confirmed the prevalence of clones harbouring cancer-associated mutations even within normal tissue. Here, we provide a succinct overview of the current state of understanding of the role of cell competition in the progression of tumours, spanning both Drosophila and mammals. Providing a comprehensive review is becoming progressively challenging due to the swift pace of advancements in this field, a recognition of its potential importance.

Cell competition represents a promising avenue in the realm of cancer therapy: similar to immunotherapy, harnessing cell competition offers a potential solution to combat drug resistance by amplifying the body’s own defences. For example, promoting the ‘winner’ status of normal cells holds the promise of acting systemically throughout a patient’s body to mitigate metastatic spread. However, this approach will require a better understanding of the precise molecular and cellular mechanisms governing competition dynamics among and between tumour and normal cells. Moreover, we will need to understand how the entire body will respond to altering these processes. Addressing these issues, coupled with a deeper mechanistic understanding, can serve as the foundation for developing a new generation of precisely targeted therapies that harness a key body defence mechanism.

To bring cell competition-based therapies to the clinics, a deeper understanding of the molecular and cellular mechanisms governing cell competition in cancer will be required. One challenge is the difficulties of studying cell competition mechanisms, which can be logistically difficult in mammalian models. With advances in patient-based spatial transcriptomics, improvements in spatially controlled CRISPR/Cas9 genome editing provides a path forward in assessing local clonal differences in vivo, in turn assessing candidate therapeutic targets. Ideally, this will include manipulating several genes in both the transformed and, independently, the wild-type cells, providing a platform to test therapeutic approaches within tumours’ complex and diverse landscapes. Leveraging cell competition as a therapeutic approach—alone or as adjunct therapy—represents an appealing opportunity to enhance cancer treatments, overcome drug resistance, and improve patient outcomes.
